# Comparison of the Curative Efficacy of Hemivertebra Resection *via* the Posterior Approach Assisted With Unilateral and Bilateral Internal Fixation in the Treatment of Congenital Scoliosis

**DOI:** 10.3389/fsurg.2022.821387

**Published:** 2022-04-01

**Authors:** Jun-Ting He, Fu-Yun Liu, Wei-Ming Hu, Jing-Jing Liu, Bing Xia, Xue-Qiang Niu, Xin-Wei Li, Yu-Feng Zhao

**Affiliations:** Department of Pediatric Orthopedics, The Third Affiliated Hospital of Zhengzhou University, Zhengzhou, China

**Keywords:** congenital scoliosis, hemivertebra resection, posterior approach, unilateral internal fixation, bilateral internal fixation

## Abstract

**Objective:**

This study aimed to compare the curative efficacy of hemivertebra resection *via* the posterior approach assisted with unilateral and bilateral internal fixation in the treatment of congenital scoliosis (CS).

**Methods:**

In this study, 29 children (15 males and 14 females), who underwent hemivertebra resection *via* the posterior approach and received internal fixation from November 2005 to September 2018, were analyzed retrospectively. The age of these patients ranged from 0.9 to 15 years, with an average of 3.8 years. The follow-up duration ranged from 2 to 12.3 years, with an average of 5.7 years. The patients in group A received unilateral internal fixation, and those in group B received bilateral internal fixation. The operation duration, bleeding volume, and complications during the operation, as well as the Cobb angles of scoliosis and kyphosis before and after the operation and at the last follow-up, were compared between the two groups.

**Results:**

In group A, the operation duration was 207.4 ± 54.5 min, and the bleeding volume was 215.3 ± 75.4 ml; in group B, the operation duration was 249.5 ± 51.0 min, and the bleeding volume was 291.3 ± 115.6 ml (*P* < 0.05). The Cobb angles of segmental scoliosis, segmental kyphosis, cephalic compensatory curve, and caudal compensatory curve were significantly improved in the two groups after operation and at the last follow-up (*P* < 0.05). The post-operative correction rate of the scoliosis Cobb angle was 67.2% in group A and 79.5% in group B; and the difference was statistically significant (*P* < 0.05). At the last follow-up, the correction rate of the scoliosis Cobb angle was 72.7% in group A and 76.2% in group B (*P* > 0.05). After the operation and at the last follow-up, the correction rates of kyphosis were 83.1 and 79.6% in group A and 71.8 and 65.5% in group B (*P* > 0.05).

**Conclusion:**

Hemivertebra resection *via* posterior approach with unilateral internal fixation can also achieve the effect of bilateral internal fixation in the treatment of CS. It is able to preserve a certain degree of contralateral spinal growth potential and is a feasible method.

## Introduction

Congenital scoliosis (CS) is a spinal deformity caused by abnormal vertebral growth in the 4th−6th weeks of fetal development. Hemivertebra is the most common type of vertebral development disorder, accounting for ~46% of cases. According to the relationship between hemivertebrae and upper and lower vertebrae, CS can be divided into three types: fully segmented hemivertebra, partially segmented hemivertebra, and non-segmented hemivertebra (1, 2). CS deformity in children is complex and even serious at birth, and develops continuously with growth; therefore, most scholars advocate early surgical treatment to block the progress of spinal deformity (3, 4). Surgical treatment methods include *in situ* fusion, convex epiphyseal block, hemivertebra resection, and spinal fusion. A hemivertebra resection *via* the anterior–posterior approach and *via* the posterior approach are commonly used due to these procedures' ability to directly remove teratogenic factors. At present, a one-stage hemivertebra resection *via* the posterior approach combined with bilateral pedicle screw internal fixation as the treatment of CS is often reported (5–8), but there are few studies on this treatment method including unilateral internal fixation. This study was designed to compare the curative efficacy of hemivertebra resection *via* the posterior approach assisted with unilateral and bilateral internal fixation in the treatment of CS.

## Materials and Methods

### General Information

In this study, 29 children with CS who underwent hemivertebra resection *via* the posterior approach and internal fixation from November 2005 to September 2018 were enrolled. The inclusion criteria included: (1) single hemivertebra deformity; (2) Cobb angle greater than 30° or increased by 5° every year; (3) no previous history of spinal internal fixation; (4) follow-up duration of ≥2 years; and (5) complete clinical data. Exclusion criteria included: (1) multiple hemivertebrae deformities; (3) a previous history of spinal internal fixation; (3) follow-up duration of <2 years; and (4) clinical data missing or incomplete. According to the fixation method, the patients were divided into the unilateral internal fixation group (group A, 14 cases) and the bilateral internal fixation group (group B, 15 cases).

Group A: There were 7 males and 7 females in this group. All the pedicles were poorly developed on the opposite side of the hemivertebra. The age of these patients ranged from 1 to 11.4 years, with an average of 3.7 ± 2.8 years. Hemivertebra position: Hemivertebra was in thoracic vertebrae in 9 cases, in lumbar vertebrae in 3 cases, and in sacral vertebrae in 2 cases. There was completely segmented hemivertebra in 12 cases and partially segmented hemivertebra in 2 cases. Overall, 8 cases (57.1%) were complicated with a tethered spinal cord, and 7 cases (50%) included a longitudinal fissure of the spinal cord, among which 6 cases underwent spinal cord surgery during that period of time. There were 4 patients (28.6%) with syringomyelia, 4 patients (28.6%) with thoracic deformity, and 2 patients (14.3%) with urinary system deformity, while 1 patient had a right abdominal wall hernia, 1 patient had right foot syndactyly, and 1 patient had left equinovarus. Group B: There were 8 males and 7 females in this group. The age of these patients ranged from 0.9 to 15 years, with an average of 3.9 ± 4.3 years. Hemivertebra position: Hemivertebra was in thoracic vertebrae in 10 cases, in lumbar vertebrae in 5 cases. There was completely segmented hemivertebra in 11 cases and partially segmented hemivertebra in 4 cases. Overall, 5 cases (33.3%) were complicated with a tethered spinal cord, and 1 case (6.7%) included a longitudinal fissure of the spinal cord, among which 5 cases underwent spinal cord surgery in that period of time. Overall, 3 patients (20%) had thoracic deformity, 1 patient had bilateral equinovarus, and 3 patients had rib absence. This study was conducted in accordance with the declaration of Helsinki and approved by the Ethics Committee of the Third Affiliated Hospital of Zhengzhou University. Written informed consent was obtained from the guardians of all participants.

### Pre-operative Preparation

The type and severity of the deformity were evaluated before the operation by an anterior and lateral x-ray of the whole spine and the left and right bending position. Computed tomography (CT) three-dimensional reconstruction of the malformation segment and whole-spinal cord magnetic resonance imaging (MRI) were carried out to determine the location and type of hemivertebra and whether there was spinal cord nervous system disease as well as to formulate the operation plan, screw placement depth, and direction. Three-dimensional CT to determine the contralateral pedicle of the hemivertebra or even the vertebral body with poor development, use unilateral internal fixation. Urinary ultrasound, residual urine volume measurement, pulmonary function test, and cardiac color Doppler ultrasound were carried out to determine the deformities of other systems and evaluate the patient's tolerance to the operation.

### Surgical Technique

All patients underwent a one-stage hemivertebra resection *via* the posterior approach. They were laid in a prone position under general anesthesia, and intraoperative electrophysiological monitoring electrodes were then placed. Routine disinfection was performed, and surgical drapes were placed. A longitudinal incision was made along the corresponding segment of the hemivertebra in the posterior median line to expose subperiosteal segments that needed to be fused up and down the hemivertebra. Intraoperative fluoroscopy was performed to position the hemivertebrae and pedicles. In group A, the contralateral lamina was slightly exposed, and the intervertebral joints were not exposed. In group B, the bilateral sides were exposed. The upper zygapophysial joint of the upper vertebra and the lower zygapophysial joint of the lower vertebra on the fixed side needed to be protected. According to the preoperative design, the vertebral body was placed in the upper and lower fixed segments of the hemivertebra (group A was unilaterally fixed, and group B was bilaterally fixed, pedicle screws were preferred, and laminar hooks could be considered for those with underdeveloped pedicles).

The posterior spinous process, lamina, transverse process, upper and lower facet processes, and partial pedicle of the hemivertebra were removed. If the hemivertebra was in the thoracic vertebrae, the proximal ribs were exposed. Additionally, the transverse costal joint and costal vertebral joint were separated, and the posterior corner of the rib to the small head of the rib was removed, cautiously avoiding damage to the parietal pleura. Careful efforts were made to protect the spinal cord and nerve root during operation. The hemivertebrae and the range of the resected vertebrae were completely resected according to the preoperative design. The epiphyseal plate and intervertebral disc were separated along the pedicle of the adjacent upper and lower normal vertebral bodies with a bone knife, and the hemivertebrae and their upper and lower intervertebral disks were gradually removed to the cancellous bone of the normal adjacent vertebrae, during which focus was placed on preserving the anterior longitudinal ligament and cleaning the cartilage surface of the adjacent normal vertebral lamina terminalis to facilitate bone graft fusion. The pre-bent connecting rod was placed and rotated. Then, in group A, a compression fixation pedicle screw was placed at the convex side (in group B, the convex side was pressurized, and the concave side was extended) to gradually correct the deformity, paying attention to correcting kyphosis. Close observation of neurophysiological changes during screw placement and correction was essential, and an intraoperative wake-up test was carried out when necessary. Under C-arm fluoroscopy, when the position of screws and rods was correct and the orthopedic effect of scoliosis was satisfactory, autogenous and allogeneic bone materials were placed for bone grafting, drainage tubes were placed, and the incision was sutured layer by layer, ending the operation. The post-operative wake-up test showed that the activities of both lower limbs were stable. The drainage tube was removed as soon as possible within 2–3 days after operation. The braces were worn for activity 2 weeks after operation, and the children were routinely asked to wear the braces for 6 months.

### Statistical Analysis

Data were statistically analyzed using the SPSS 21.0 software. Measurement data were expressed as mean ± standard deviation (x ± SD). Count data were expressed as cases and percentages (%). Data were compared using a *t*-test. The inspection level was α = 0.05. A *P*-value of <0.05 was considered statistically significant.

## Results

The summary on hemivertebra resection levels and follow-up results for all patients was shown in Table 1. In group A, the operation duration was 150–385 min (average: 207.4 ± 54.5 min), and the bleeding volume was 60–380 ml (average: 215.3 ± 75.4 ml). In group B, the operation duration was 135–330 min (average, 249.5 ± 51.0 min), and the bleeding volume was 100–600 ml (average: 291.3 ± 115.6 ml). The differences in the operation duration and bleeding volume between the two groups were statistically significant (*P* < 0.05, Table 2). The follow-up duration of all cases ranged from 2 to 12.3 years, with an average of 5.7 ± 3.0 years. The number of fused vertebral bodies was 2–6, with an average of 3.2 ± 1.1. In group A, one patient had transient nerve injury and recovered after receiving a methylprednisolone sodium succinate injection during the operation, and one patient had pleural effusion after the operation, with improved symptoms after closed thoracic drainage. No patients had serious complications, such as pseudojoint, internal fixation loosening, or serious infection. In group B, one patient had pleural effusion after the operation and improved after symptomatic treatment; one patient did not wear a brace 1 month after the operation, had pedicle cutting, and underwent reoperation. One case underwent reoperation due to incomplete resection of hemivertebra after operation.

**Table 1:** The summary on hemivertebra resection levels and follow-up results for all patients.

**Case**	**Age**	**Sex**	**operation time (min)**	**Blood loss (ml)**	**Hemivertebra level**	**Pedicle screws**	**Hook**	**Segmental curve (°)**			**Segmental kyphosis (°)**			**Compensatory cranial curve (°)**			**Compensatory caudal curve (°)**		
								**Pre**	**Post**	**Follow-up**	**Pre**	**Post**	**Follow-up**	**Pre**	**Post**	**Follow-up**	**Pre**	**Post**	**Follow-up**
1	3 years 3 months	M	200	250	T11-T12	1	1	52	20	25	15	−5	4	6	0	10	15	1	5
2	1 years 7 months	M	150	60	T11-T12	1	1	40	9	8	25	4	8	10	5	3	22	0	3
3	1 years 8 months	M	175	140	L2-L3	0	2	35	7	6	6	−16	−14	6	5	20	15	1	1
4	1 years 4 months	F	205	169	T9-T10	1	1	38	14	1	25	15	5	12	8	3	15	7	3
5	1 years	F	190	190	L3-L4	0	2	51	4	−2	33	5	10	13	10	3	1	1	0
6	1 years 4 months	M	185	175	T9-T10	2		40	10	4	19	10	5	10	2	4	8	4	10
7	6 years 6 months	F	385	280	T5-T6	2		48	21	10	20	10	13	23	2	5	4	4	6
8	2 years	M	200	200	T8-T9	0	2	35	19	10	12	1	2	22	5	15	13	2	8
9	7 years 2 months	F	185	275	T10-T11	3		30	12	20	−2	−2	−1	10	15	30	22	3	15
10	2 years 9 months	F	205	380	T6-T7	6		50	22	21	38	21	25	35	30	30	5	4	11
11	4 years	M	180	250	T8-T9	5		55	11	10	30	9	10	40	16	10	15	2	1
12	11 years 5 months	F	228	220	L5-S1	4		20	10	12	90	20	26	20	18	7	1	0	0
13	2 years 6 months	F	200	180	L5-S1	2		20	5	1	25	15	10	40	6	13	0	0	0
14	4 years 10 months	M	215	245	L5-S1	2		22	7	6	−30	−20	−25	46	5	5	0	0	0
15	1 years 10 months	M	220	100	T9-T10	6		48	8	4	40	16	17	9	6	5	27	15	14
16	3 years 7 months	F	330	360	T11-T12	6		68	15	20	35	20	35	5	2	2	8	3	2
17	1 years 2 months	M	200	230	T11-T12	4		45	18	7	40	9	16	8	6	5	6	5	0
18	4 years 5 months	F	238	180	T8-T9	6		35	13	8	15	3	5	16	6	6	15	4	9
19	1 years 6 months	M	190	260	T7-T8	6		35	12	17	17	10	11	8	6	5	18	12	13
20	14 years	M	310	600	T10-11	5		22	4	5	49	25	35	10	3	3	14	2	3
21	1 years 3 months	M	235	245	T12-L1	6		35	10	15	18	11	12	9	−5	2	8	4	3
22	1 years 1 months	F	290	270	L2-L3	6		46	5	9	9	−9	−16	5	1	−2	8	0	3
23	15 years	F	250	230	T3-T4	4		46	16	20	37	18	20	10	8	7	15	4	5
24	3 years	F	285	400	L2-L3	6		33	5	10	20	16	20	4	−9	−3	2	2	−1
25	3 years 7 months	F	300	390	T6-T7	6		49	8	15	30	11	13	10	3	3	10	4	11
26	2 years 6 months	F	235	250	L1-L2	6		49	6	3	11	−5	−8	30	7	3	16	8	2
27	2 years 7 months	M	265	240	T11-T12	6		35	2	5	16	3	5	6	0	2	8	0	1
28	11 months	M	135	325	T10-T11	6		35	3	4	21	5	8	9	8	2	7	−5	−3
29	2 years	M	260	290	L2-L3	4		27	2	3	18	9	15	14	−8	2	8	5	−3

*Unilateral internal fixation, case 1–14; Bilateral internal fixation, case15–29; M, male; F, female; Pre, preoperative; Post, postoperative; Follow-up, last follow-up*.

**Table 2:** Comparison of operation duration, intraoperative bleeding volume and correction rate of scoliosis between the two groups.

**Group**	***N***	**Male**	**Female**	**Age (year)**	**Operation duration (min)**	**Intraoperative bleeding volume (mL)**	**Postoperative correction rate of scoliosis**	**Follow-up correction rate of scoliosis**
Unilateral internal fixation group	14	7	7	3.7 ± 2.8	207.4 ± 54.5	215.3 ± 75.4	67.2 ± 13.3	72.7 ± 22.4
Bilateral internal fixation group	15	8	7	3.9 ± 4.3	249.5 ± 51.0	291.3 ± 115.6	79.5 ± 11.6	76.2 ± 13.4

*Comparison of operation duration and intraoperative bleeding volume between the two groups P <0.05; comparison of post-operative correction rate of scoliosis between the two groups P < 0.05; comparison of follow-up correction rate of scoliosis between the two groups P > 0.05*.

**Figure 1:**
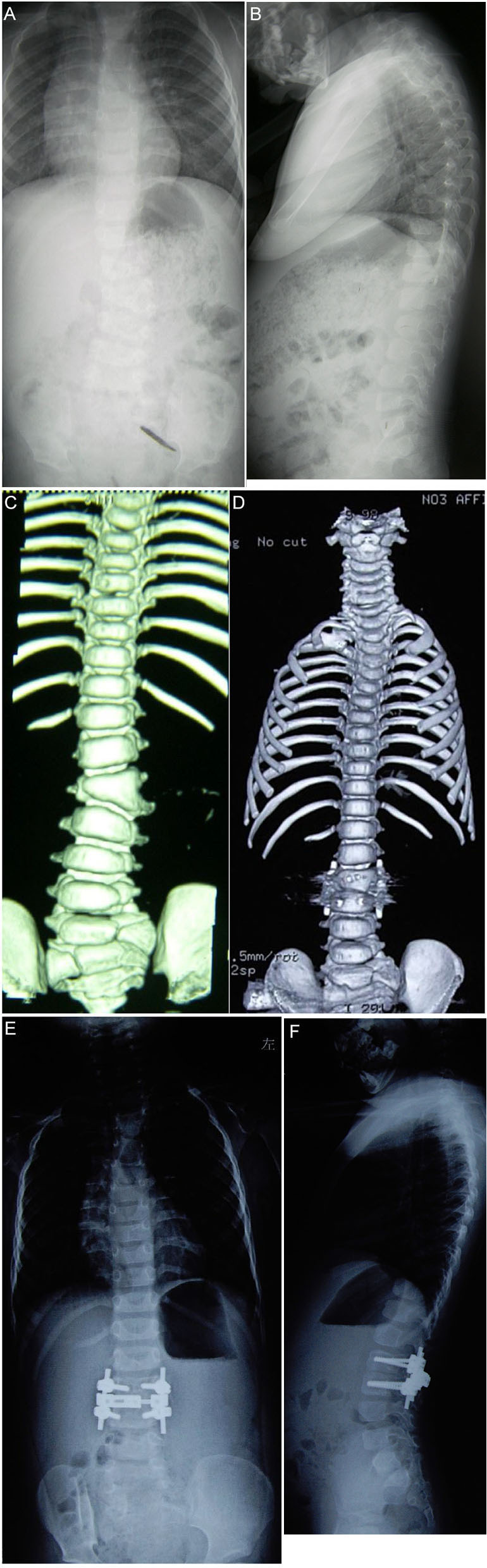
The child is a 2-year-old boy. Preoperative x-ray [**(A)** coronal position, **(B)** sagittal position]and computed tomography (CT) **(C)** reveal that local deformity left of the L3 hemivertebra is obvious, and hemivertebra resection *via* the posterior approach assisted with single segment bilateral pedicle screw internal fixation is performed. Postoperative CT **(D)** reveals that the osteotomy space is stable and the orthopedic effect is good. An x-ray 2 years after the operation revealed that the orthopedic effect was good, and there was no significant loss during follow-up ([**(E)** coronal position, **(F)** sagittal position].

**Figure 2:**
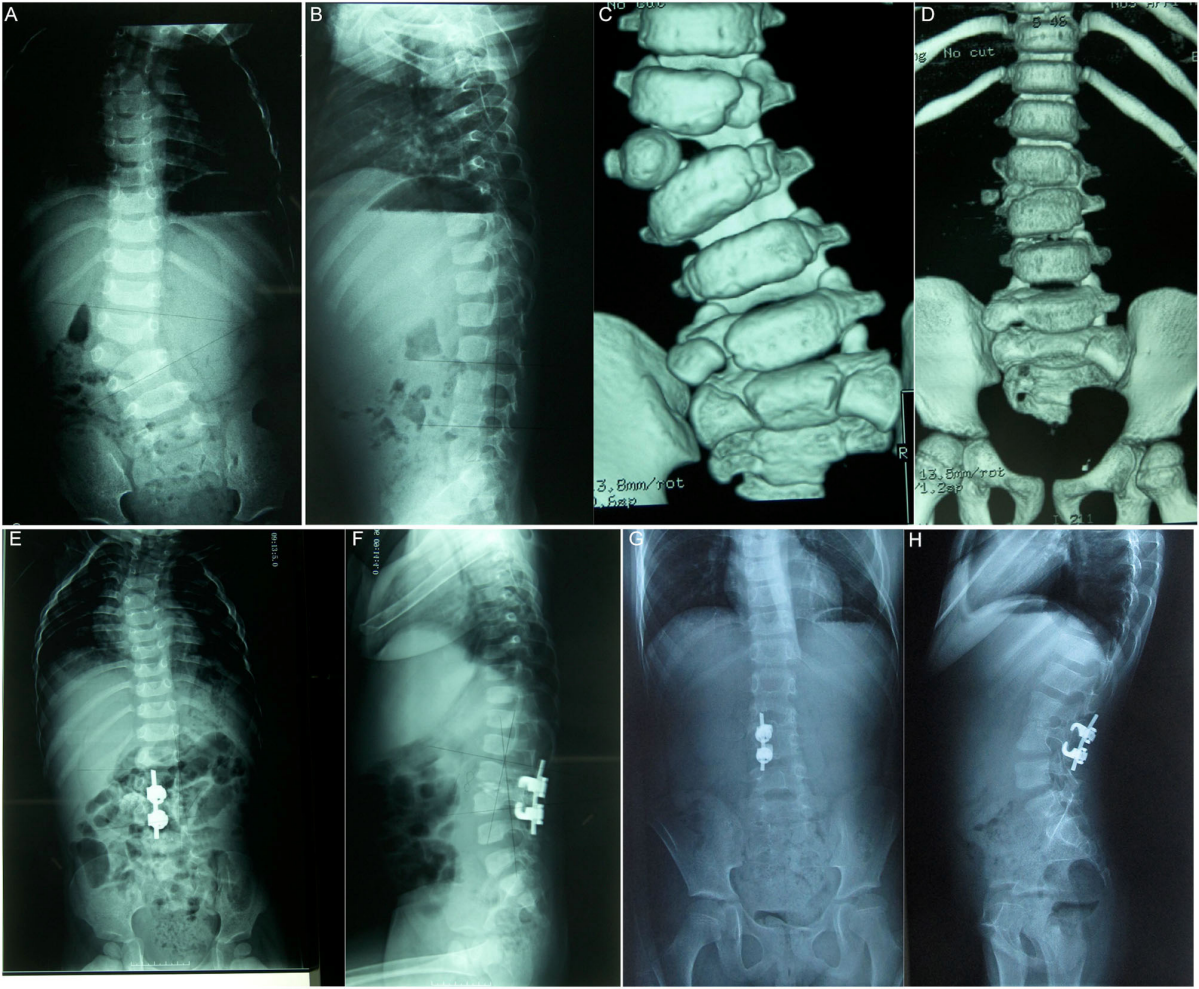
The patient is a 20-month-old boy. The right L3 is a completely segmented hemivertebrae, and the preoperative Cobb angle of scoliosis is 35°. Hemivertebra resection *via* the posterior approach of grade 5 osteotomy assisted by unilateral hook rod internal fixation was performed **(A–C)**. Post-operative CT **(D)** reveals that the osteotomy space is stable, and the orthopedic effect is good. The postoperative Cobb angle of scoliosis is 7°, the orthopedic effect was good 8 years after the operation, and there was no significant loss of angle at follow-up **(E,G)**. The sagittal position reveals that preoperative kyphosis turns into physiological lordosis **(B,F,H)**.

**Figure 3:**
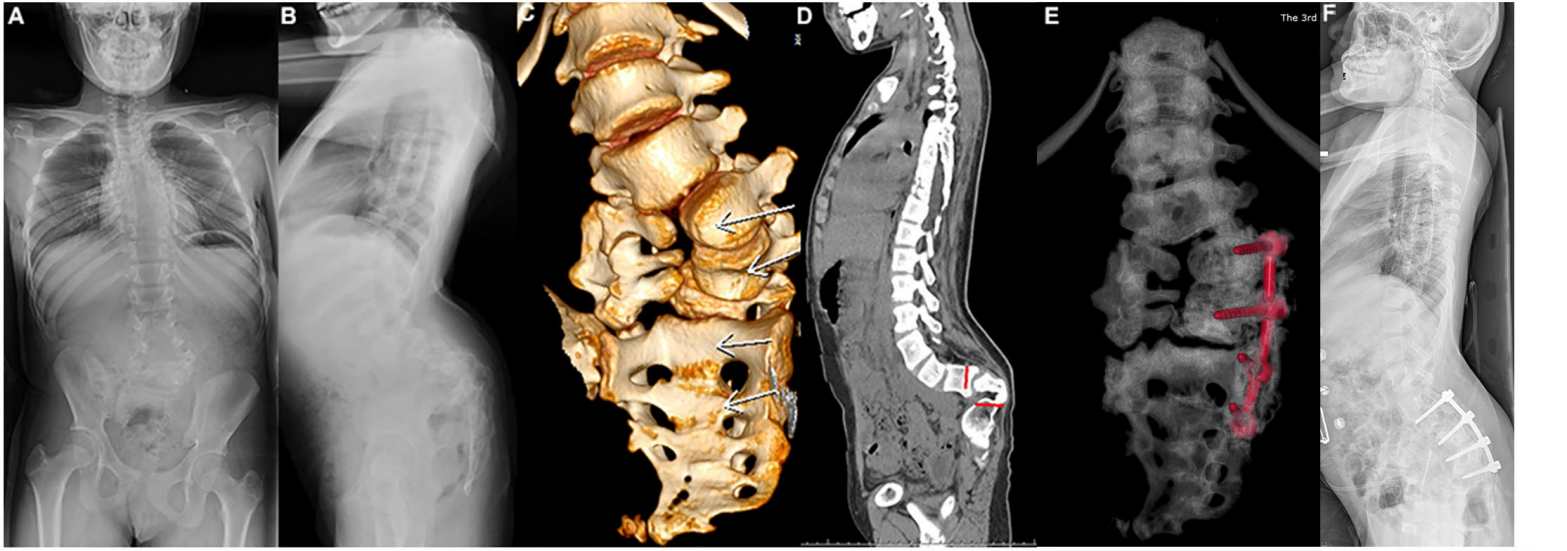
Female aged 11 years, L5–S1 hemivertebra, lumbosacral kyphosis, due to the poor development of the pedicle of the right vertebral body of L4-6, the Schwab grade 6 osteotomy of the left hemivertebral body between L5S1 and the internal fixation of the left L4, L5, S1 and S2 screw rods were performed. Preoperative x-ray [**(A)** coronal position, **(B)** sagittal position] and computed tomography (CT) **(C,D)**, CT and X-rays after surgery **(E,F)**.

**Figure 4:**
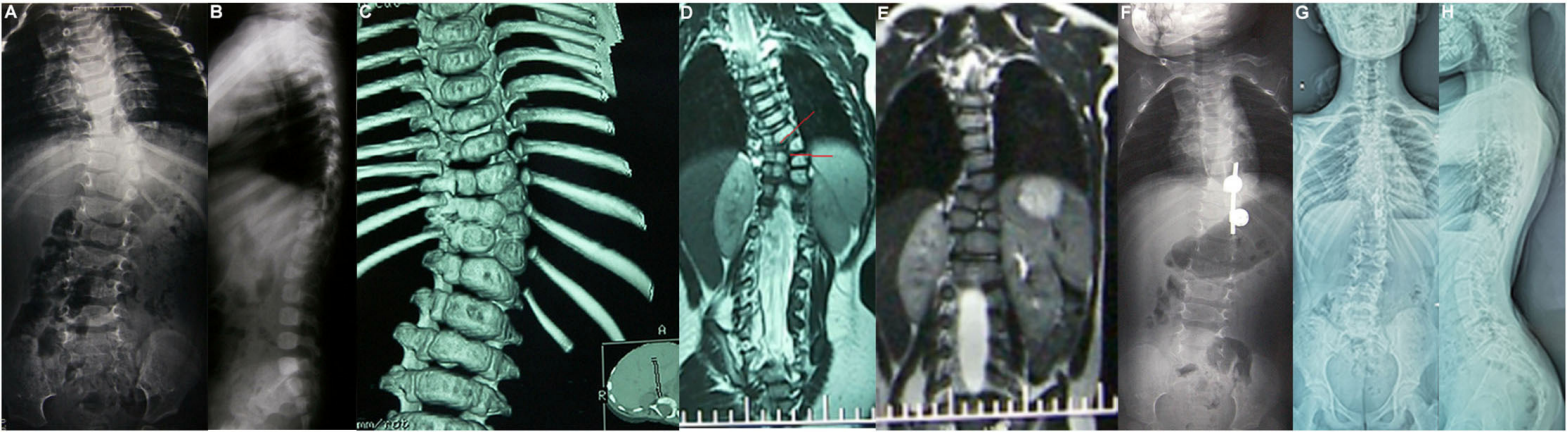
Female aged 1 years 4 months, T9–T10 hemivertebra; T8-12 bone bridge complicated with type II SCM, one-stage posterior T9–10 hemivertebra Schwab grade 5 osteotomy, T9-10 nail hook internal fixation, preoperative x-ray **(A,B)**, preoperative CT **(C,D)**, postoperative CT **(E)**, postoperative x-ray **(F)** 1 year after operation, and the X-ray display was further improved 12 years after the last follow-up **(G,H)**.

There was no significant difference in the Cobb angles of segmental scoliosis and segmental kyphosis before the operation between the two groups (*P* > 0.05). The Cobb angles of segmental scoliosis, segmental kyphosis, cephalic compensatory curve, and caudal compensatory curve were significantly improved in the two groups after the operation and at the last follow-up (*P* < 0.05, Table 3). The postoperative correction rate of the scoliosis Cobb angle was 67.2% in group A and 79.5% in group B, and the difference was statistically significant (*P* < 0.05). At the last follow-up, the correction rate of the scoliosis Cobb angle was 72.7% in group A and 76.2% in group B, and the difference was not statistically significant (*P* > 0.05). After the operation and at the last follow-up, the correction rates of kyphosis were 83.1 and 79.6% in group A and 71.8 and 65.5% in group B, meaning the differences were not statistically significant (*P* > 0.05). Typical cases are presented in Figures 1–4.

**Table 3:** Comparison of the orthopedic effects between two groups.

**Item**	**Unilateral internal fixation group**			**Bilateral internal fixation group**		
	**Before operation**	**After operation**	**At follow-up**	**Before operation**	**After operation**	**At follow-up**
Segmental scoliosis (°)	38.3 ± 12.1	12.2 ± 6.1	9.4 ± 8.0	40.5 ± 11.2	8.5 ± 5.3	9.7 ± 6.2
Segmental kyphosis (°)	21.9 ± 26.0	4.8 ± 12.3	5.6 ± 13.3	25.1 ± 12.3	9.5 ± 9.1	12.5 ± 13.4
Cephalic compensatory curve (°)	20.9 ± 13.9	9.1 ± 8.1	11.3 ± 9.4	10.2 ± 6.3	2.3 ± 5.6	2.8 ± 2.7
Caudal compensatory curve (°)	9.7 ± 8.0	2.1 ± 2.1	4.5 ± 4.9	11.3 ± 6.2	4.2 ± 4.8	3.9 ± 5.4

*Comparison of the Cobb angles of segmental scoliosis, segmental kyphosis, cephalic compensatory curve and caudal compensatory curve in the two groups between after the operation and at the last follow-up with those before the operation P < 0.05; comparison of scoliosis and kyphosis before the operation between the two groups P > 0.05; comparison of scoliosis and kyphosis at the last follow-up between the two groups P > 0.05*.

## Discussion

For scoliosis and kyphosis caused by hemivertebra deformity, as deformity progresses rapidly, and the compensatory curve progresses to structural curve with growth and development, the effect of conservative treatment is poor, so early surgical intervention is very important (5–7). The goal of surgery is, in the case of short segment fusion, to maintain spinal function, correct spinal deformity, and maintain the normal development of the spine and thorax as much as possible (1, 2). The operation method should be selected according to the location and type of hemivertebra, the progress rate of scoliosis, the deformity of other systems, and the age of the patient. Hemivertebra resection can remove teratogenic factors from the root and achieve a good orthopedic effect (8). Hemivertebra resection was first reported by Royle in 1928. Limited by the conditions at that time, only part of the lamina and articular process were fused without internal fixation. Some scholars reported that simple hemivertebra resection without internal fixation could achieve the desired effect. Report (3). If the hemivertebra is not completely resected, and there is a residue after the operation, the deformity will gradually become aggravated with the growth of hemivertebra. With the improvement of spinal surgery technology and the maturity of pedicle screw technology, scholars have reported performing a one- or multiple-stage hemivertebra resection *via* an anterior–posterior approach assisted with internal fixation, or a hemivertebra resection *via* the posterior approach assisted with bilateral internal fixation (9–12). Scholars in China reported that 432 patients underwent a one-stage hemivertebra resection *via* the posterior approach for CS, stating the correction rates of scoliosis and kyphosis were 81.1 and 73.9%, respectively, and complications occurred in 57 cases (11). It has also been reported that after hemivertebra resection *via* the posterior approach assisted with short segment internal fixation, the correction rates of segmental scoliosis and kyphosis were 65.9 and 61.9%, respectively (12). In this study, the average correction rates of scoliosis and kyphosis in the bilateral internal fixation group were 79.5 and 76.2%, respectively. These rates were consistent with those reported in literature.

Posterior hemivertebral resection with bilateral internal fixation is the most common method for early treatment of congenital spinal deformities (13–15). However, the authors found that the development of the pedicle in the fixed segment was poor in some children, especially the pedicle on the opposite side of the hemivertebra, and the development of the vertebral body on the opposite side of the hemivertebra was also poor. For such children, the author used unilateral internal fixation as Group A, compared with group B, the post-operative correction effect was better with bilateral fixation, but there was no statistical difference at the last follow-up, indicating that unilateral fixation can also achieve the effect of bilateral fixation. The absence of fusion on the contralateral vertebral body resulted in further improvement. Feng et al. reported that for a hemivertebra resection *via* the posterior approach assisted by unilateral internal fixation, the average operation time was 153.7 min and the average blood loss was 214.2 ml; the correction rates of segmental scoliosis and kyphosis were 61.5 and 75.3%, respectively; and the correction rates of the cephalic and caudal compensatory curves were 46.1 and 54.5% (16). Group B did not use laminar hooks; group A had 9 laminar hooks, which were chosen because of poor pedicle development in young children, but there was no pedicle screw stabilization when laminar hooks were used during the operation. Most use pedicle screw fixation. In the author's group B, there was 1 case of pedicle screw cutting fracture after surgery, and the analysis was that it was not caused by plaster external fixation, short bracing time or no firm fixation. Group A did not have such complications, which may be because the parents thought that unilateral fixation was insufficient. Firm, the external fixation of the brace is firm and wears for a long time. Marco Crostelli (7) and other scholars all used pedicle screw internal fixation, and there was no cutting fracture complication after plaster fixation for an average of 4 months after surgery. It is suggested that pedicle screw fixation after hemivertebra resection is the most ideal internal fixation method. In this study, compared with the bilateral internal fixation group, the unilateral internal fixation group had a shorter operation time, less intraoperative bleeding volume. At the same time, the correction rates of scoliosis and kyphosis in the unilateral internal fixation group were consistent with those of previous reports. Unilateral internal fixation preserves the growth ability of the contralateral side (the concave side) and limits the growth of the internal fixation side (the convex side), which provides a good opportunity for the subsequent long-term correction of spinal deformities. In addition, a unilateral pedicle screw (lamina hook) internal fixation is suitable for patients with unilateral pedicle dysplasia or without a pedicle that is unable to be fixed bilaterally (Figure 3). Xue and Zhao adopted hemivertebra resection *via* the posterior approach assisted with unilateral internal fixation fusion to observe the growth of the unfused concave side of the spine (17). It is considered a safe, effective, and minimally invasive method, and it can reduce concave growth block, which is an early correction method worth recommending.

In conclusion, posterior hemivertebra resection and unilateral internal fixation in the treatment of congenital scoliosis can also achieve the effect of bilateral internal fixation and retain a certain degree of growth potential of the contralateral spine. It is a feasible method and is worthy of further study in clinical work. However, due to the late development of unilateral internal fixation, there have been relatively few clinical cases, so it still needs long-term follow-up observation. When choosing the surgical scheme, we should strictly grasp the surgical indications in combination with the patient situation.

## Data Availability Statement

The original contributions presented in the study are included in the article/supplementary material, further inquiries can be directed to the corresponding author.

## Ethics Statement

The studies involving human participants were reviewed and approved by the Ethics Committee of the Third Affiliated Hospital of Zhengzhou University. Written informed consent to participate in this study was provided by the participants' legal guardian/next of kin.

## Author Contributions

J-TH and F-YL: conception and design of the research. J-TH and W-MH: acquisition of data and writing of the manuscript. X-QN and X-WL: analysis and interpretation of the data. J-JL and Y-FZ: statistical analysis. F-YL: obtaining financing. F-YL and BX: critical revision of the manuscript for intellectual content. All authors read and approved the final draft.

## Funding

This study was supported by Congenital Scoliosis Genetics and Proteomics Research Project (No. LHGJ20190384).

## Conflict of Interest

The authors declare that the research was conducted in the absence of any commercial or financial relationships that could be construed as a potential conflict of interest.

## Publisher's Note

All claims expressed in this article are solely those of the authors and do not necessarily represent those of their affiliated organizations, or those of the publisher, the editors and the reviewers. Any product that may be evaluated in this article, or claim that may be made by its manufacturer, is not guaranteed or endorsed by the publisher.
